# Large Metasurface Aperture for Millimeter Wave Computational Imaging at the Human-Scale

**DOI:** 10.1038/srep42650

**Published:** 2017-02-20

**Authors:** J. N. Gollub, O. Yurduseven, K. P. Trofatter, D. Arnitz, M. F. Imani, T. Sleasman, M. Boyarsky, A. Rose, A. Pedross-Engel, H. Odabasi, T. Zvolensky, G. Lipworth, D. Brady, D. L. Marks, M. S. Reynolds, D. R. Smith

**Affiliations:** 1 Center for Metamaterials and Integrated Plasmonics. Duke University, Box 90291, Durham, NC 27708, USA; 2 Department of Electrical and Computer Engineering, Duke University, Durham, NC 27708, USA; 3 Department of Electrical Engineering, University of Washington, Seattle, 98195, USA; 4 Evolv Technology, 200 West Street, Waltham, MA 02451, USA; 5 Department of Computer Science and Engineering University of Washington, Seattle, WA 98195, USA

## Abstract

We demonstrate a low-profile holographic imaging system at millimeter wavelengths based on an aperture composed of frequency-diverse metasurfaces. Utilizing measurements of spatially-diverse field patterns, diffraction-limited images of human-sized subjects are reconstructed. The system is driven by a single microwave source swept over a band of frequencies (17.5–26.5 GHz) and switched between a collection of transmit and receive metasurface panels. High fidelity image reconstruction requires a precise model for each field pattern generated by the aperture, as well as the manner in which the field scatters from objects in the scene. This constraint makes scaling of computational imaging systems inherently challenging for electrically large, coherent apertures. To meet the demanding requirements, we introduce computational methods and calibration approaches that enable rapid and accurate imaging performance.

Microwave and millimeter wave (mmW) radio frequency signals (1–300 GHz) can penetrate many optically opaque materials, allowing visualization of hidden objects. Economical radio hardware in this regime supports coherent measurements from a large number of sub-apertures to realize three dimensional (3D) image reconstruction. However, it is non-trivial to realize *spatially* coherent measurements across the large aperture necessary to achieve the resolution required for applications such as security screening, through-wall imaging, automotive radar, machine vision, and medical diagnosis^1^,^2^,^3^,^4^,^5^,^6^,^7^,^8^. As the demand for mmW imaging increases, low-cost and robust approaches are being sought that can be deployed in large volume.

Archetypical approaches to mmW imaging have relied on either (a) sampling an aperture with a dense array of sources for beam forming, as in active electronically scanned antennas (AESAs)^9^, or (b) mechanically scanning a transceiver over the aperture, as in synthetic aperture radar (SAR) systems^10^,^11^. The former approach yields fast acquisition times but with expensive hardware, while the latter approach has inherently slow acquisition time. Both approaches result in a sampling of the aperture at roughly the Nyquist limit (half the free space wavelength), making either beam forming possible or allowing fast Fourier transform (FFT) techniques to be applied for image reconstruction.

The limitations associated with conventional approaches can be circumvented by adopting a more abstract perspective of the measurement process. As we abandon conventional antenna elements and Nyquist sampling of the full aperture, the computationally simple relationships between measured data and image reconstruction no longer exist. Fortunately, modern computational imaging (CI) schemes^12^ provide the mathematical foundation for advanced imaging systems that can take advantage of arbitrary measurement modalities. Leveraging continual advances in computing power, CI approaches have become increasingly viable, relaxing hardware constraints and enabling alternative aperture architectures to be explored.

Across the electromagnetic spectrum, from microwave to x-ray, and even in the acoustic regime, demonstrations of CI approaches are numerous in the literature^13^,^14^,^15^,^16^,^17^,^18^,^19^. A particularly relevant example, coded apertures, has enabled the development of single pixel imaging systems at infrared, terahertz (THz) and x-ray wavelengths^20^,^21^,^22^ where detector arrays may be prohibitively expensive. In these systems, light reflected from a scene is passed through a set of masks, each of which has a spatially varying transparency, and is then focused to a detector. Scene information is thus multiplexed across many non-orthogonal measurements, with the image reconstructed using more general CI algorithms. The variable mask plus detector can be thought of as a large aperture antenna that either measures or produces complex radiation patterns, or measurement modes. Coded aperture systems trade-off complex modes and processing to minimize the need for expensive detectors.

The CI concept has also been used to take advantage of frequency-diversity, with several demonstrations of metasurface apertures that produce distinct mode patterns as a function of frequency. Such apertures can acquire scene information using only a frequency sweep from a single source, without moving parts^23^.

Metasurfaces, a more practical outgrowth of metamaterials research^24^,^25^,^26^, are appealing for imaging applications for several reasons. Propagating waves through a single metamaterial layer circumvents many of the problematic aspects associated with volumetric metamaterials. Moreover, the enormous flexibility associated with metasurface designs provides tremendous opportunities for the development of apertures that optimally leverage CI algorithms. The frequency-diverse metasurface aperture is attractive because it can be fabricated at mmW wavelengths using standard printed circuit manufacturing, leading to an extremely low-cost and low-profile form factor.

## Results

### Millimeter wave imaging system

**Figure 1:**
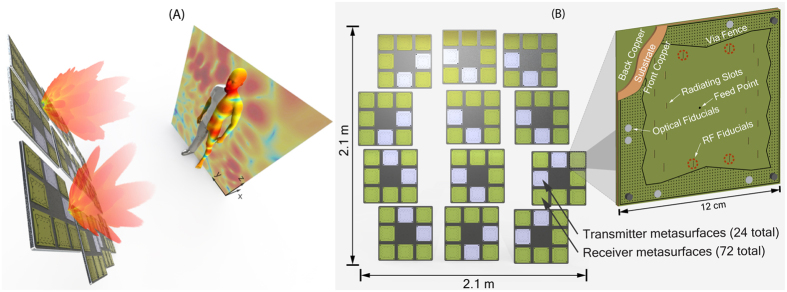
(**A**) Frequency swept measurements are acquired from each combination of the 24 transmit (blue) and 72 receive (green) metasurface panels in the imaging system. The measurements probe the scene with complex radiation patterns, as shown here projected on a mannequin target at 1 m. RF radiation passes through weak dielectric materials, such as clothing, but reflects off the body, metallic objects, and high dielectrics. The frequency-diverse response of the PCB based metasurfaces, shown in (**B**), provide a large set of distinct radiation patterns for image reconstruction.

The imaging system demonstrated here is based on an array of metasurface panels, as indicated in Fig. 1. Each metasurface panel is constructed of low-loss, copper clad printed circuit substrate (Rogers 3003, Rogers Corporation). The top and bottom bounding copper layers, plus a via fence at the periphery of the board, define a 2D irregularly shaped dielectric cavity, as illustrated in Fig. 1(B). A back mounted coaxial connector feeds a cylindrical coplanar waveguide mode into the cavity at an off-center point. Subwavelength irises, etched into the top copper surface, sample the waveguide mode and transmit or receive radiation. The spatially varying waveguide modes within the irregular circuit board cavity feed the irises, which in turn produce distinct radiation patterns that vary as a function of the driving frequency^27^,^28^,^29^,^30^.

Because each panel can only support a limited number of frequency-diverse measurements, the composite imaging system consists of 24 transmit (Tx) panels and 72 receive (Rx) panels (12 cm × 12 cm). The metasurface panels are distributed over a 2.1 m × 2.1 m aperture, as shown in Fig. 1. The metasurface sub-aperture panels are grouped into modules for mechanical convenience, which in turn are distributed on an irregular grid; the removal of periodicity avoids aliasing in the reconstructed images. In this configuration, a given Tx panel is repeatedly excited by a frequency sweep, with measurements taken sequentially on all of the Rx panels. The total number of measurements available from the system is 24 × 72 × *N*_f_, where *N*_f_ is the number of frequencies measured. Measurements are taken from each pair of Tx/Rx panels using a switch-based signal distribution network. The system is driven by a single custom homodyne RF transceiver (radio) that sweeps across the K-band (17.5–26.5 GHz) in discrete frequency steps. The received in-phase and quadrature-phase data is sampled by the radio hardware and sent to a host PC for image reconstruction. The average noise floor of the radio is −100 dB, allowing for the measurement of weak return signals from small objects or dielectrics with permittivity close to air.

Maximizing the set of useful frequencies measurements, *N*_f,_ implies maximizing the diversity of the radiated fields, which in turn requires optimization of the geometry of the metasurface. To assess the imaging capacity of the panels, we perform a plane-wave decomposition of the radiative modes and a mapping of their spatial frequencies (k-space, as described in the Supporting Online Material - SOM). For the frequency-diverse panels, the distribution of the irises determines the accessible Fourier space, while the variability of the modes in the cavity—across the frequency band—determines the specific Fourier components that are sampled at each measurement mode^31^.

Investigation of the k-space support led to the Mills Cross design^32^. The irises have a slot shape and are oriented horizontally, at the top and bottom of the transmitter metasurface panels; and vertically, at the left and right of the receiver metasurface panels, as shown in Fig. 1(B). This sparse distribution provides the same k-space support as a densely spaced iris layout. The use of minimally redundant antenna distributions, such as Golay patterns, is well established in radio astronomy where it reduces hardware requirements^33^. Here, by minimizing the number of radiation channels, we statistically increase the phase accumulation along the different scattering paths inside the cavity, which in turn, manifests as increased mode diversity between frequency steps (widening the impulse response). The loaded quality factor, Q, of a panel encapsulates this mode diversity and is straight-forwardly measured^34^. A higher Q statistically ensures mode diversity and maximizes the number of distinct measurement modes available for imaging. The number of useful frequency measurements for a set of frequency-diverse panels can be estimated as *N*_f_ = *QB*/*f*_*0*_, where *B* is the bandwidth and *f*_0_ is the center frequency of the bandwidth^31^. The number of distinct frequency measurements, *N*_f_, determines the maximum dimension of the measurement space that can be measured, and hence the number of spatial components that can be observed. For the metasurface panel pair, ideally *N*_f_ ≈ (*N*_iris_)^2^, where *N*_iris_ is the number of radiating irises on each Tx and Rx metasurface panel. Designing the metasurface panels requires balancing the material loss, size, the number of radiation irises, and the backend hardware requirements, to achieve the above equality. The optimal Q of the panels was designed to be *Q* ≈ 330, giving approximately *N*_f_ = 135 useful frequency measurements for the 10 cm × 10 cm cavity embedded in the panels (*N*_f_ = 100 was used in experiments). A distribution of *N*_iris_ = 16 radiating irises on the panels (slightly above the predicted value) was found to be a compromise between maximizing spatial frequency sampling and maintaining the necessary signal-to-noise in the system^32^. The metasurface panels have an average radiation efficiency of *η* ≈ 30%.

### Experimental imaging

**Figure 2:**
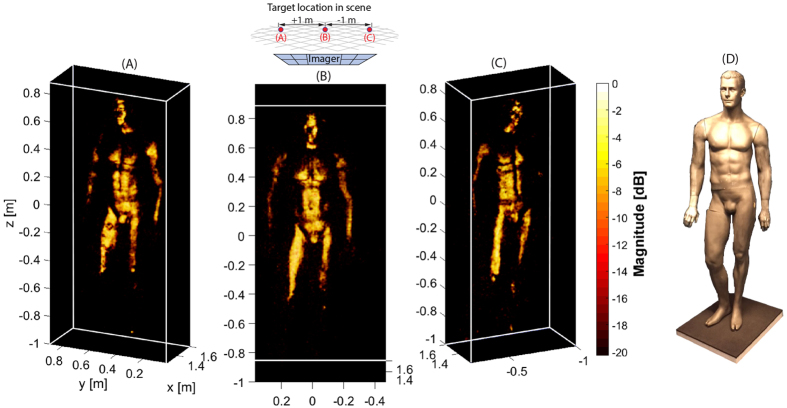
Least squares image reconstruction of a mannequin (covered with conductive paint): (**A**) Mannequin position offset to the left, Y = 0.6 m; (**B**) Mannequin at center, Y = 0 m; (**C**) Mannequin offset to the right, Y = −0.6 m. (**D**) Visible light photograph of the mannequin.

In Fig. 2, we present experimental images of a full human sized mannequin at three different locations in the scene (with varying perspectives to demonstrate the 3D nature of the images). The mannequin is painted with conductive nickel paint to approximate the high reflectivity characteristics of human skin at mmW. Fine feature detail of the mannequin (at the diffraction limit) is clearly observable in these fully 3D images. We note that conductive targets at mmW often exhibit strong specularity due to their high conductivity and low surface roughness (with respect to the illumination wavelength), regardless of the imaging approach used. This reduces the viewing angle, in contrast to the diffuse scattering more commonly observed at optical wavelengths.

To reconstruct the images shown in Fig. 2, a complete characterization of the spatial field distribution corresponding to each of the Tx and Rx panels is necessary, as is an accurate model for object scattering. The importance of this model cannot be overstated; model accuracy dictates image fidelity in a CI system. For the scattering model, as is typical in mmW imaging, we apply the first Born approximation, which assumes the electric field reflected by a volume element is directly proportional to the incident field, or *E*_ref_(**r**) = *f*(**r**)*E*_inc_(**r**). This approximation neglects both material dispersion and assumes isotropy—both good approximations at mmW bands. With the scene space divided into discrete volume elements, or voxels, the relationship between the field measurements **g**_i_ and reflectivity values **f**_i_ takes the form of a matrix equation **g** = **Hf** + **n**, where **H**_ij_**∝E**_i_^Tx^**E**_j_^Rx^ are the elements of an *M*x*N* measurement matrix and **n** is noise in the system. That is, the measurement matrix (**H**) elements are proportional to the field from the Tx panel at a given point in space, **E**^Tx^, multiplied by the field from the Rx panel at the same point, **E**^Rx^. In the absence of noise and with a complete set of orthogonal measurement modes, the scene reflectivity could be found by simple matrix inversion. However, for modes that exhibit correlation, as in our system, and with the number of measurements either under or over-sampling the scene, **H** cannot be inverted directly. The strategy for image reconstruction, then, is to estimate the image by solving 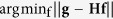 for the reflectivity vector **f** that minimizes the expression. We use a least squares (LS) algorithm here with an appropriate regularizer^35^.

### Image resolution and field of view

The resolution of the metasurface imager can be understood in the general context of SAR, for which the range resolution relates to the operational bandwidth, *δ*_r_ = *c*/2*B*, while the cross-range resolution is determined by the aperture size, *δ*_c_ = *λ*_min_*D*/*L*, where *λ*_min_ is the wavelength, *D* is the distance to the target, and *L* is the aperture width^10^. These estimates suggest resolution limits of *δ*_c_ = 5.4 mm and *δ*_r_ = 16.7 mm. The achieved resolution of the metasurface imager, however, is dependent on the actual sampling of the k-space as previously discussed. Critically, even if the aperture is sampled sparsely, reconstructions over a subset of the volume can still achieve diffraction limited resolution. For a specific imaging configuration, the singular value decomposition (SVD) of **H** provides a useful means of assessing the system’s imaging capacity. A nearly flat singular value spectrum signifies greater orthogonality of the measurements modes, while a decaying spectrum signifies redundancy in the measurements modes.

**Figure 3:**
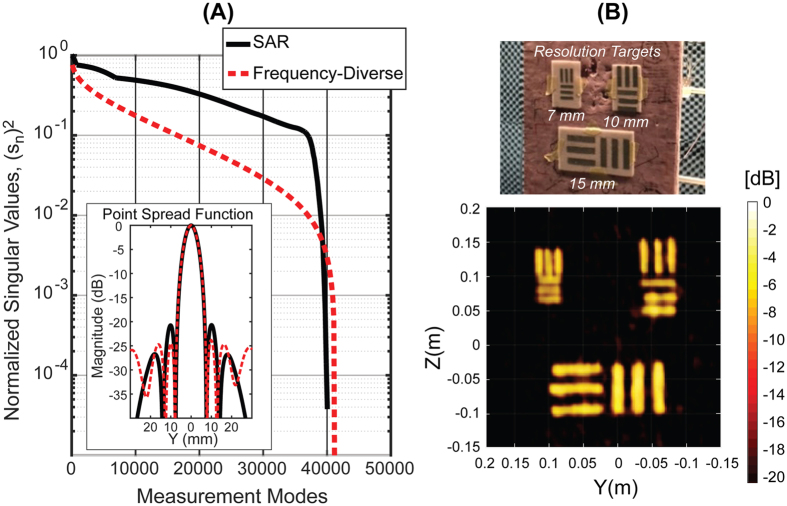
(**A**) The simulated singular value spectrum over a 2 m^2^ cross range scene slice with N_f_ = 100 frequency sampling points over the K-band (17.5–26.5 GHz), compared with a SAR system having a comparable number of measurement modes as described—operating at two frequencies (17.5 GHz, 26.5 GHz). The Simulated PSF response (Matched Filter) for both systems is also shown; (**B**) Experimental images (LS reconstruction) of 7 mm, 10 mm, and 15 mm resolution targets showing the ability to clearly resolve the targets at the diffraction limit.

We use the SVD of the measurement matrix as a means of quantitatively assessing the cross-range measurement capacity of the system, considering a cross-range slice of 2 m^2^ in the scene at a distance of 1 meter. This area exceeds the estimated average cross-section of a person^36^, roughly 0.75 m^2^. Figure 3(A) shows the singular value spectrum of the frequency-diverse metasurface system prototyped here, and for comparison, a similarly sized monostatic SAR system. The SAR system utilizes two frequencies (17.5 GHz, 26.5 GHz) with aperture sampling at the midband Nyquist limit (7 mm), such that it has nearly the same number of measurement modes as the metasurface system. The measurement matrix for the SAR system possesses a nearly flat singular value spectrum as shown in Fig. 3(A). For the metasurface system, the non-orthogonality of the measurement modes results in a decaying slope; however, with sufficient signal-to-noise ratio and appropriate algorithmic approaches, it is possible to acquire a majority of the information available in the scene. This is confirmed with an analysis of the system point spread function (PSF)^37^, shown inset in Fig. 3(A). For this analysis the simple adjoint operator is used, i.e. f = H*g (where H* is the conjugate of **H)**. In this manner we assess the PSF independent of the noise level. We obtain a cross-range resolution that approaches the diffraction limit—6.1 mm for the metasurface system and for the SAR system. Experimentally we have confirmed the resolution limits of the metasurface system, using resolution targets, as shown in Fig. 3(B). The critical comparison here is that both systems exhibit similar imaging resolution for a similar number of measurement modes. Leveraging frequency diversity thus eliminates the need for mechanical scanning or a vast number of switched elements to reach the ~80,000 Nyquist sampled measurements across the entire aperture, as would be needed for SAR imaging. There are only 96 switching channels for the metasurface system, for which the swept frequency measurements can be made extremely fast using current continuous wave radio hardware. This suggests a technical approach that can achieve fast imaging rates to rival electronic SAR systems but at much lower component costs comparable to mechanically scanned SAR systems.

For potential 3D imaging applications, such as personnel screening, the imaging volume can be physically large. A human can fill a rectangular volume of ~2.5 million voxels when sampled at the diffraction limit. Fortunately, natural images often conform to simplifying priors, such as scene sparsity. This knowledge is utilized in compressive algorithms to extract an image from an underdetermined measurement set^38^,^39^. Here, a simple physical prior is appropriate and computationally inexpensive. The system is augmented with an optical structured light sensor (Kinect, Microsoft) to identify the target and reduce the extent of the reconstruction region. As an initial step in the imaging sequence, information about the target received from the Kinect sensor is used to build a voxel cage over the surface of the target; the reflectivity is then reconstructed at these voxel locations only, significantly reducing the computational effort.

**Figure 4:**
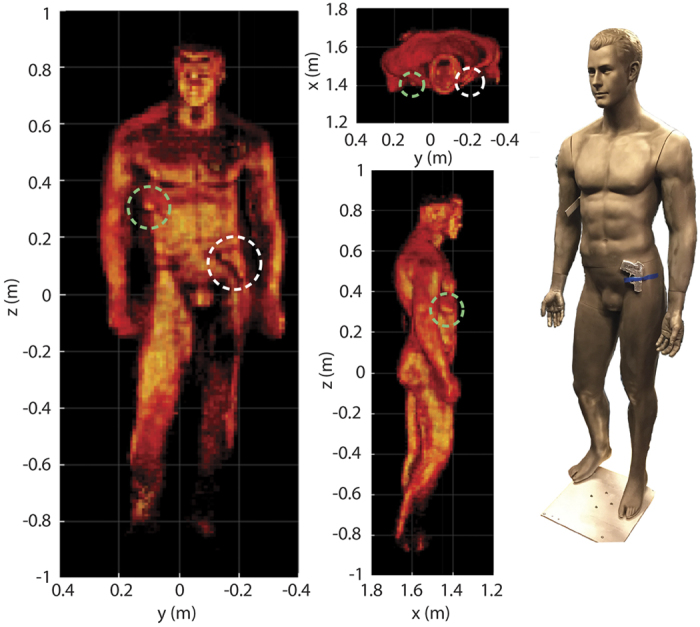
Multiple experimental images can be stitched together to reveal the full detail of the mannequin and thereby overcome the limited specular view observed for any single pose. This approach enhances the detection of threat objects, such as the gun phantom (right side of the body, white dashed line) and knife phantom (left armpit, green dashed line) shown here. This image is composed of images taken of a mannequin as it was rotated through 5 degree increments.

Because the Kinect sensor can locate the target dynamically, the imaging region is not tightly constrained; rather, the target can be positioned anywhere within the field-of-view of the system. If the data acquisition and reconstruction time is suitably fast, targets can be imaged while in motion, allowing multiple images to be collected with non-redundant information. Such images can be stitched together to provide enhanced resolution, or to resolve image artifacts due to specularity and model error. In Fig. 4, we demonstrate the prospects for enhanced imaging, forming a composite image from a sequence of reconstructed images of a mannequin rotated in 5 degree increments (details and a rotating movie of the stitching can be found in the SOM). We see that the stitched image exposes nearly all surface detail of the mannequin as compared with any of the single images shown in Fig. 2.

**Figure 5:**
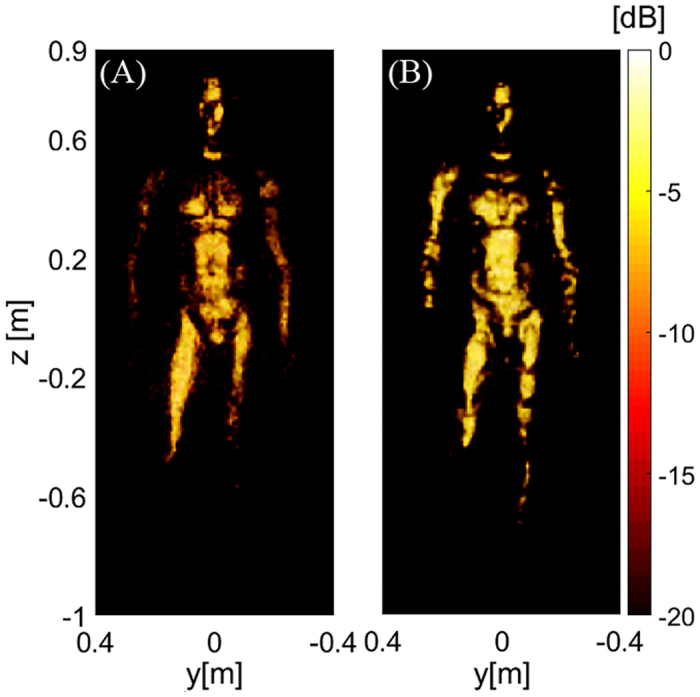
(**A**) Measured vs. (**B**) simulated reconstruction of a mannequin. The simulated target was generated from a 3D model captured using a structured light measurement. Image quality and specular profile shows good qualitative agreement, thus validating the experimental reconstruction.

To assess the system performance in comparison to its theoretical capacity, we first image the mannequin target using the Kinect sensor, creating a stereo-lithography (STL) file that can be imported into software simulation tools we have developed. A voxel cage is then built around the STL model exactly as done in the experiment, and the imported fields from the panels are used to illuminate the digitized mannequin. The mannequin voxels are given reflectivity values corresponding to the actual painted mannequin (*f*_target_~0.8), and the image reconstructed from the virtual measurements. Figure 5 shows the excellent agreement obtained between the simulated and measured mannequin target.

Because the simulated measurements implicitly assume perfect alignment between the fields and the virtual panels, the simulated reconstruction represents the best achievable image reconstruction fidelity. The experimental reconstructions are obtained by taking the actual measurements from the system and incorporating the alignment procedure described in the Methods section. If the alignment is not achieved to within a fairly tight tolerance, the reconstructed image is rapidly destroyed. The agreement between the simulated and measured mannequin target confirms that alignment errors have been minimized.

**Figure 6:**
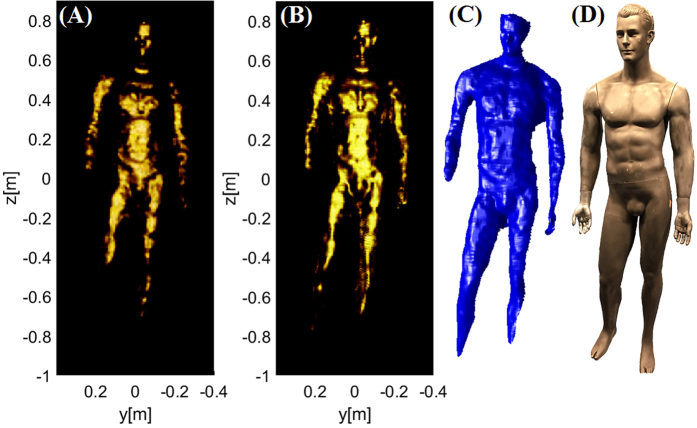
A simulated reconstruction of the mannequin using (**A**) the metasurface system and (**B**) comparable SAR system, with parameters described previously in Fig. 3, is shown. (**C**) The 3D STL model was captured using a structured light measurement of an actual mannequin as shown in (**D**).

The imported STL model can also be used to compare the reconstruction capabilities of the metasurface system versus the comparable SAR system previously described in Fig. 3. The metasurface system is seen to be of similar quality to that of the SAR reconstruction in Fig. 6, despite the sparse spatial sampling of the aperture. We note that imperfections in the imported model (due to limitations of the structured light sensor) limit the absolute quality of either reconstruction simulation. None-the-less, for a similar number of allotted measurements, the study confirms that the metasurface approach can achieve comparable image quality to traditional SAR approaches.

## Discussion

Frequency-diverse metasurfaces provide a uniquely flexible platform for exploiting CI capabilities. This approach can readily be scaled to higher frequencies, where the benefits in minimizing active components and their associated costs are even more compelling. Rather than relying strictly on frequency-diversity and switching networks, dynamically reconfigurable metasurfaces can be implemented that would enable inexpensive imaging systems with reduced frequency bandwidth requirements^40^,^41^,^42^,^43^,^44^. Whether static or dynamic, metasurface architectures are poised to revolutionize RF aperture design, and represent an exciting paradigm for imaging sensors in the mmW and THz regimes.

## Methods

### Characterization of the measurement modes

The quality imaging observed in Figs 2, 3, 4, 5, 6 demands accurate knowledge of the fields in the scene. In experimental implementation, it is difficult to obtain precise field patterns for the metasurface apertures from either analytical models or full-wave simulations, at the level required for an accurate measurement matrix. We thus obtain a characterization of the fields using near-field scanning (NFS), in which a probe antenna measures the radiated field over a plane close to the aperture. Because of manufacturing tolerances, the fields from each of the 96 panels must be separately measured and stored^45^,^46^. From these near-field scans, the fields can be numerically propagated everywhere over the field-of-view (FOV) by application of the dyadic Green’s function propagator^47^, allowing a complete characterization of the fields over the imaging volume and hence of the measurement matrix.

### System alignment

Once the field values are determined by near-field scans, they must be brought into coincidence with the actual physical locations of the panels within the final layout. To facilitate alignment across the full 2.1 m × 2.1 m aperture, we incorporate four additional radiating irises into the metasurface panels, in isolated locations, to act as alignment fiducials, as shown Fig. 1(B). Their physical locations are determined in relation to the near-field scans, via field propagating and search methods. Optical fiducials are also integrated onto the surfaces of the panels. Once the panels are bolted into the frame, their locations and orientations are determined by stereophotogrammetry (3D MAXShot, CREAform). Finally, the appropriate transformations from the NFS to the imaging coordinate system are determined, allowing exact orientation of the measured radiation patterns of the metasurfaces, regardless of their placement and orientation.

### Image reconstruction

Once the metasurface panels have been aligned and characterized, the measurement matrix must be determined throughout the scene. Even with the optical sensor constraining reconstruction to an envelope of voxels within +/−4 cm of the front surface of the mannequin, the number of required voxels for diffraction-limited resolution is still large, on the order of 100,000–200,000. To facilitate a tractable reconstruction, we make use of the information that each pair of Tx and Rx panels has a predominant k-vector, and use the analytical form of the propagator to enable a partitioning of the full measurement matrix into a set of smaller sub-matrices^48^. The computation of these sub-matrices can be further parallelized using graphical processing units (GPUs), so that the equivalent required measurement matrix operations can be performed in minutes or even seconds. We apply this computational approach as a simulation tool to design, evaluate, and optimize the metasurface panels as well as the overall multi-panel aperture. We also use the computational technique for image reconstructions, importing the near-field scan data and scene measurement data.

### Phase calibration of the feeding network

In addition to the precise alignment of the physical panels with respect to their computed field distributions, it is also necessary to obtain an accurate calibration of the phase advance through the feeding network to each metasurface panel^49^. Measuring the phase offset for each Tx/Rx path directly, however, is futile due to the perturbative effects that mechanically connecting the paths together would have on the measurements. Instead, we apply an in-scene calibration approach, in which two wideband dipole antennas within the scene transmit directly to all Tx/Rx metasurface panels. The positions of these dipole antennas are localized using the stereophotogrammetric approach, such that the free-space path lengths between all the metasurface panels and the antennas are determined. The phase advance along each path is then accurately modeled in software and subtracted from the actual experimental measurement, giving the desired calibration correction. This approach can be performed periodically to counter any drift in the feeding network and radio.

## Additional Information

**How to cite this article:** Gollub, J. N. *et al*. Large Metasurface Aperture for Millimeter Wave Computational Imaging at the Human-Scale. *Sci. Rep.* **7**, 42650; doi: 10.1038/srep42650 (2017).

**Publisher's note:** Springer Nature remains neutral with regard to jurisdictional claims in published maps and institutional affiliations.

## Supplementary Material

Supplementary Video 1

Supplementary Material
