# Supplementary material for: Large Metasurface Aperture for Millimeter Wave Computational Imaging at the Human-Scale
Source: Sci Rep. 2017 Feb 20;7:42650. doi: 10.1038/srep42650 (PMC5316995; doi:10.1038/srep42650)
Supplement: Supplementary Material [file srep42650-s2.doc]

Supplementary Materials For

**Large Metasurface Aperture for Millimeter Wave Computational Imaging at the Human-Scale**

J. N. Gollub^1,5*^, O. Yurduseven^1,5^, K. P. Trofatter^1,5^, D. Arnitz^2^, M. F. Imani^1,5^, T. Sleasman^1,5^, M. Boyarsky^1,5^, A. Rose^3^, A. Pedross-Engel^2^, H. Odabasi^1,5^, T. Zvolensky^1,5^, G. Lipworth^1,5^, D. Brady^5^, D. L. Marks^1,5^, M. S. Reynolds^2,4^, and D. R. Smith^1,5^

Correspondence to: [jonah.gollub@duke.edu](mailto:jonah.gollub@duke.edu)

**This file includes:**

Supplementary Text

Figs. S1 to S13

Captions for Movie S14

Supplementary Text

**System Configuration**

This section discusses the system configuration and the imaging process

**
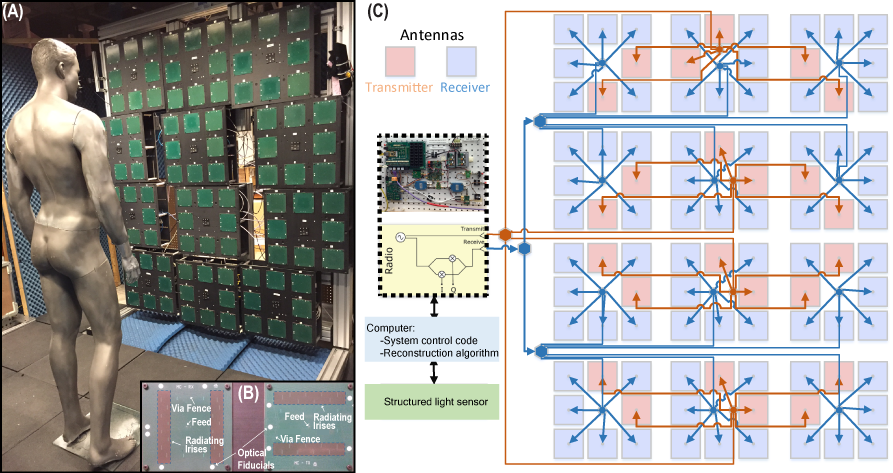
**

***Fig. S1.*** *(A) an image of the system and (B) close up image of the panels is shown. (C) Schematic detailing the position of the transmitter and receiver panels, as well as the switching network. The radio, switching, and IR sensors are all managed by a standard workstation computer.*

The system configuration consists of 24 transmit (Tx) and 72 receive (Rx) metasurface panels which are grouped into sub-modules as shown in Fig. S1. One hundred frequency measurements are acquired for each Tx and Rx combination, using a custom radio across the K-band (17.5 – 26.5 GHz), as discussed below. A switching network of coaxial K-band rated cables (Gigalink, A52FP flexible & semi-rigid) and mechanical switches (Keysight, 6-port, L7106C) provide a path from the radio to each Tx and Rx pair (24x72=1728 paths). For speed, the low-level switching and radio control are managed by an ARM Cortex-M3 microcontroller and custom-designed SPI-controlled switch control boards---which avoids the lag in speed that results from communication through the computer operating system’s overhead. The Cortex-M3 is controlled via communication with the workstation using SCPI (Standard Commands for Programmable Instruments) compatible high level start, stop, and parameter set commands. Measurement data is transferred via Ethernet. The high-level system control code is written in Matlab.

**Panel Characterization**

This section details the fabrication and characterization of the panels.

**
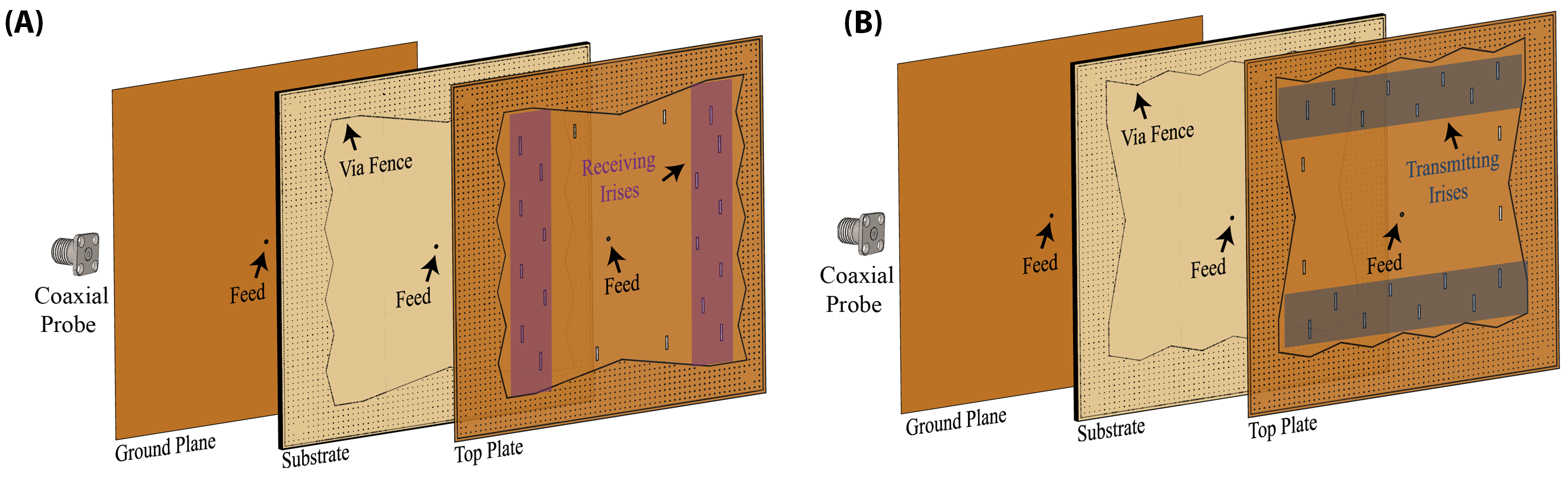
**

***Fig. S2.*** *An illustration of the layered PCB design of the metasurface panels, (A) receiver and (B) transmitter, is shown. The vias and copper surfaces are seen to confine the waveforms within the panels. A K-band rated SMA connector and its center feed wire are soldered to the back and front of the panels, respectively.*

A detailed breakout of the panels, described in the main text, is shown in Fig. S2. The panels are fabricated using low loss copper clad Rogers 3003. Fabrication is performed with standard chemical etching and copper deposition (for metallization of the vias). The size of the panel etching geometry is on the millimeter scale and can be fabricated at a standard PCB fabrication house.

The quality factor, Q, of the panels is determined from a measurement of the impulse response of the panels at their feed point (S_11_ scattering parameter). In the time domain, the response is fitted with the characteristic exponential decay, $e^{-2t/\tau}$, and $\tau$is determined. The quality factor is then determined by $Q=\tau\omega_{0}/2$, where $\omega_{0}$ is the mean angular frequency of the bandwidth. The panels were found to have a quality factor on average of *Q*=~330.


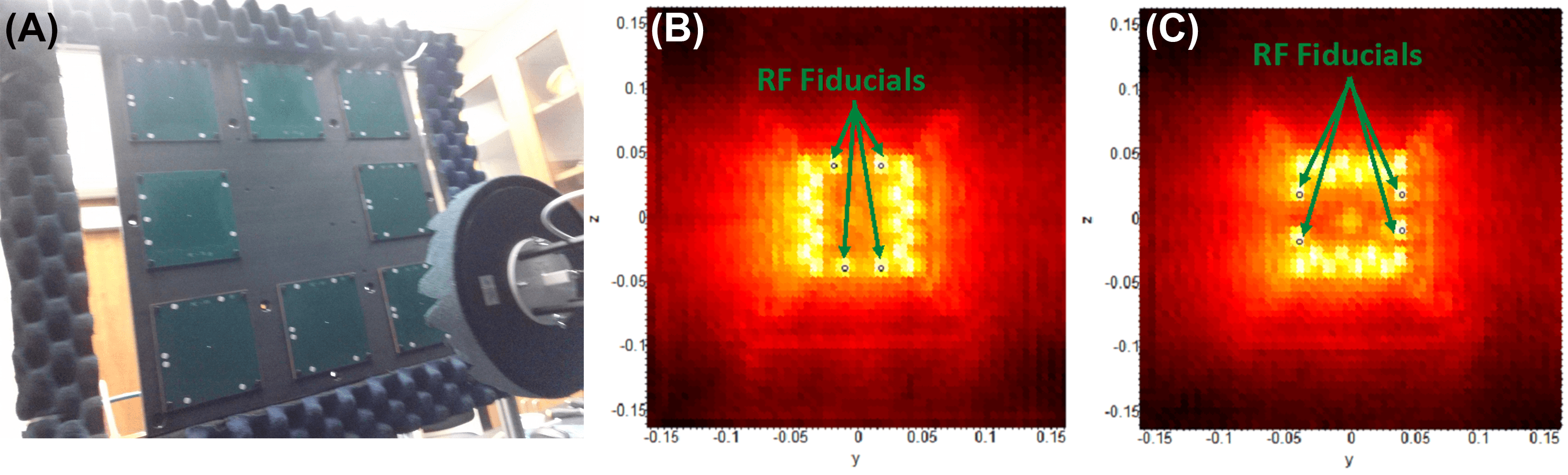


***Fig. S3.*** *(A) Near field raster scanning is performed on the panels to characterize them (using a NSI scanning system). (B-C) The near field scans (back propagated to the panel plane and summed over all frequencies) are shown. The RF fiducials, used for alignment, are seen to be spatially isolated from the other radiating elements allowing them to be localized.*

The vertically oriented slots etched into the front of the panels emit radiation with a single horizontal polarization. These fields are characterized using a low gain WR-42 waveguide probe that is raster scanned throughout a 2D planar surface in front of the panels. One hundred frequency measurements (spaced across the K-band) are taken at each sampling position, 5 mm spacing (below the cross-range Nyquist sampling rate of 5.7 mm for the maximum driving frequency of 26.5 GHz). For two characteristic Tx and Rx metasurface panels, the fields (summed over frequency) are shown in Fig. S3. Each panel is scanned at a stand-off distance of $5\lambda_{\text{min}}$(8.6 cm) across a 40 cm x 40 cm 2D plane. The near field scan serves to completely determine the radiation properties of the panel in the scene over a 60-degree angular range given standard field propagation techniques, as determined by the scan area and probing distance. Scans take ~45 minutes and all panels are scanned individually to account for any variation in their radiation signatures. We note that, given the size of the characteristic geometrical details, it should be possible to fabricate the panels with a consistent accuracy such that each set of Tx an Rx panels would exhibit identical radiation characteristics. This would require only a single Tx and Rx panel to be scanned in order to completely characterize the whole system---simplifying the deployment of the system. However, this fabrication refinement is a focus for future efforts. The radiation efficiencies of the metasurface panels were found by summing the discrete field measurements over the near field scan (NFS) plane, with an appropriate normalization to account for the NFS probe measurement efficiency. The efficiencies were found to be 28% for the Tx panels, and 34% for the Rx panels.

**
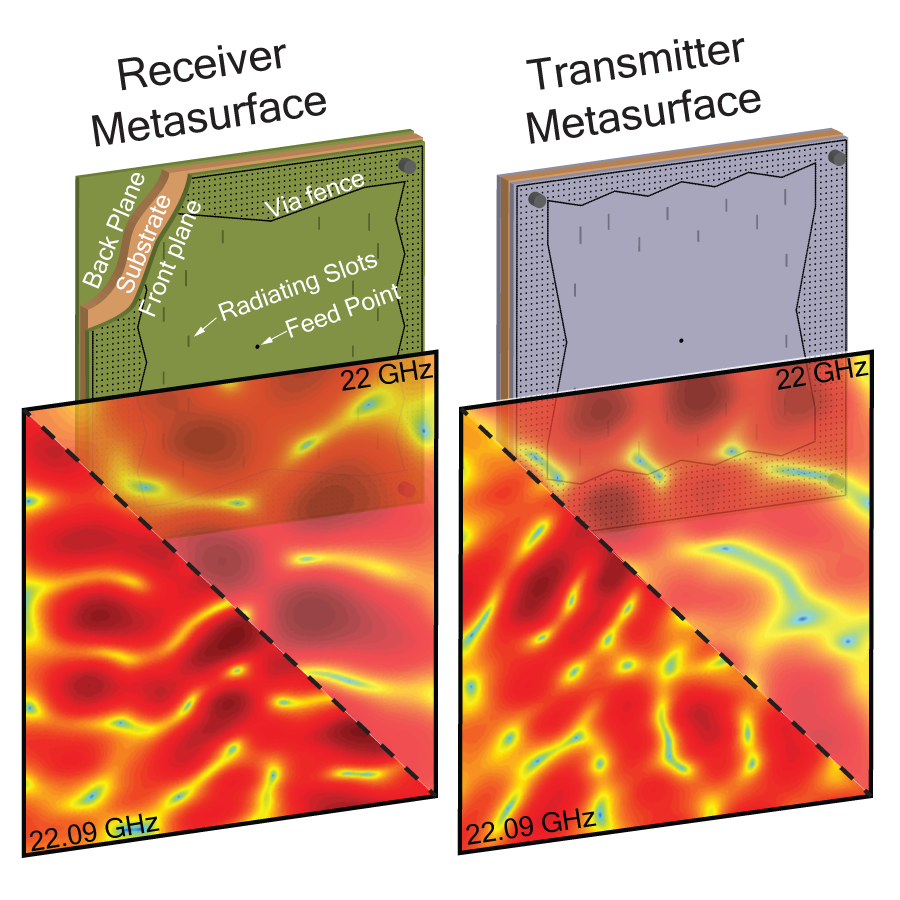
**

***Fig. S4.*** *The field profiles (propagated to 1 m) of the Tx and Rx panels are shown for two adjacent frequency sample points, 22 GHz and 22.09 GHz. The strong field diversity generated by the metasurface panels supports a convenient set of measurement modes for interrogation of the scene.*

The strong frequency diversity of the fields are observed when the near field scans are propagated to the scene, as shown in Fig. S4. The fields varying significantly between frequency steps and provide a distinct set of measurement modes for imaging.

**Panel Optimization**

This section discusses the details of the Mills Cross metasurface design.

The imaging capacity of a system is generally related to its coverage in Fourier space (or k-space). In the multistatic system considered here, the k-space support for any transmitter/receiver set is a convolution of their radiated fields, in the Fourier domain. The k-space support for the whole system is the union of these Fourier domains. Hence, optimizing the k-space support of the individual Tx/Rx pairs can generally improve the full system’s capabilities. The *individual* panels are small compared to the distance at which imagining is performed. To determine the convolution in this far-field region, it is approximately equivalent to assess the spatial convolution of the fields at the aperture. These spatial convolution (convolution of the NFS back-propagated to the panel plane) are shown in Fig. S5. At various sampling frequencies we get distinct field patterns that translate into distinct measurement modes in the image domain; when we sum all of them, we see that the “effective aperture” is uniformly and evenly sampled, signifying efficient sampling. Though this analysis is only an approximation, it suggest that the sparse Mills Cross based metasurface panel design provides effective sampling of the scene while minimizing the number of radiating elements.


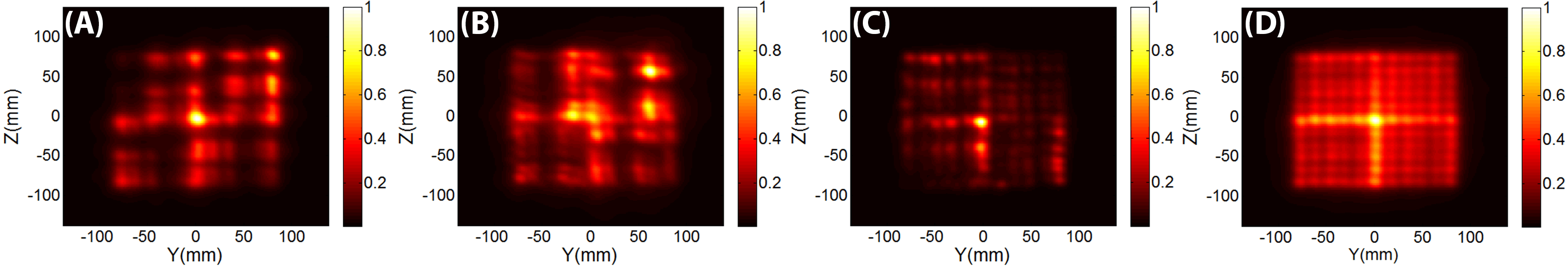
***Fig. S5.*** *The k-space support is approximately the convolution of the effective aperture of the transmitter receiver panels at the aperture plane (A) 17.5 GHz (B) 22 GHz (C) 26.5 GHz (D) all measurements superposed over the K-band (100 frequency points).*

**Radio**

This section describes the RF backend hardware and the radio system in detail.

The RF backend hardware consists of three main blocks: the RF signal distribution using Keysight L7106C 6-port RF switches, the RF transceiver (“radio”) hardware, and sampling hardware. All three blocks are controlled by a central microcontroller running custom firmware. A top-level block diagram of the system is shown in Fig. S6.The host computer interfaces with the radio using a clear-text-based SCPI-like protocol. Data is streamed back to the host in IEEE 488.2 Definite Length Arbitrary Block Response Data packets over Ethernet (UDP). Both protocols are commonly used in network analyzers and other measurement devices and are thus readily available in many programming languages. Central processor of the RF hardware is a 32-bit ARM Cortex-M3 microcontroller (Arduino Due). The microcontroller receives the SCPI commands and interfaces with the radio hardware using SPI (Serial Programming Interface Bus), and performs the real-time tasks controlling the radio.


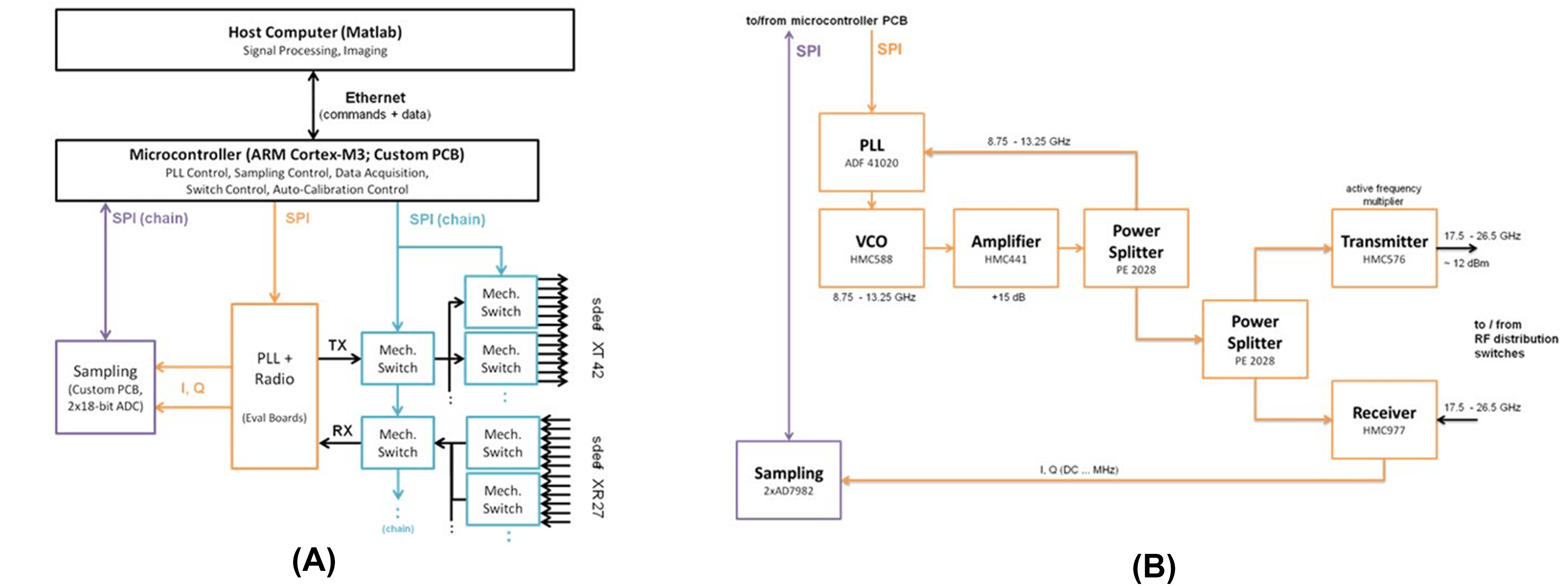


***Fig. S6.*** *Top-level block diagram of radio (A) and radio block diagram (B).*

The radio RF hardware consists of an Analog Devices ADF41020 Integer-N Phase-Locked-Loop (PLL) with a Hittite HMC588 Voltage Controlled Oscillator (VCO) generating a stepped local oscillator (LO) signal between 8.75 GHz and 13.25 GHz. A Hittite HMC441 medium-power amplifier is used to boost the LO signal, and two Pasternack PE2028 power splitters distribute the LO signal to the PLL, the transmitter, and the receiver. The transmitter consists of a Hittite HMC576 x2 active frequency multiplier, whereas the receiver consists of a Hittite HMC977 IQ downconverter with integrated x2 frequency multiplier. Transmitter and receiver form a homodyne transceiver block in this configuration, with ~12 dBm transmit power spanning 17.5 to 26.5 GHz. Sampling of the downconverted in-phase (I) and quadrature-phase (Q) channels is performed by Analog Devices ADS8861 true 16-bit ADCs using a custom-designed printed circuit board (PCB). The data is transmitted to the microcontroller via SPI and then streamed to the host PC over Ethernet using UDP data packets.


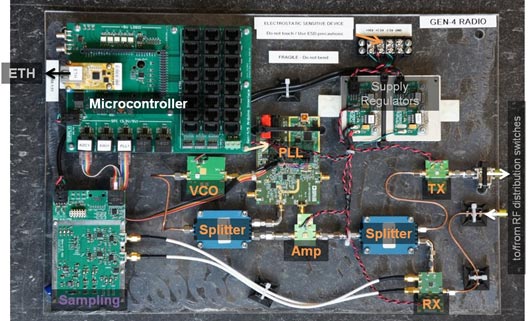


***Fig. S7.*** *Photograph of the radio and control hardware using evaluation boards for all RF components.*

Fig. S7 shows a photograph of the hardware setup. This version of the radio uses modified evaluation boards for PLL, VCO, LO amplifier, Tx and Rx. Details of the RF distribution switches and associated control boards are provided in Fig. S8. Local calibration of the radio is performed in post-processing using a single stored measurements of known calibration standards (through and isolation) composed of two 20 dB Pasternack PE7088-20 attenuators and a short Pasternack PE34735-6 semi-rigid cable connecting TX and RX ports.


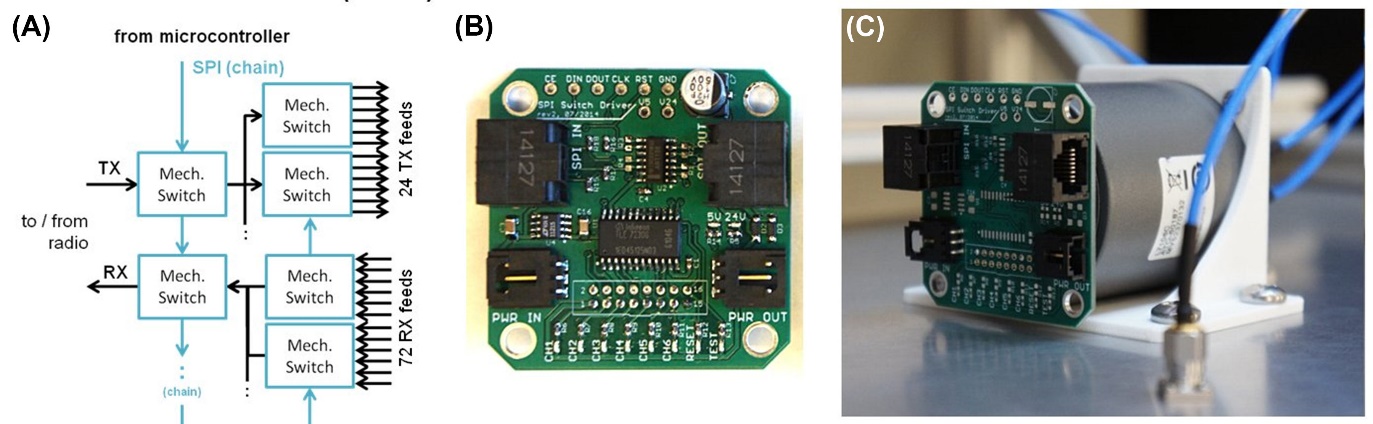


***Fig. S8.*** *(A) RF Distribution Switch Block diagram, (B) custom switch PCB and (C) image of PCB on Keysight L7106C 6-port RF switch.*

Fig. S9 shows the accuracy of the radio compared to a Keysight N5527A network analyzer (VNA). The figure shows multiple |S21| measurements of two daisy-chained PE7088-30 30dB attenuators with 60 dB total attenuation compared to the same measurement taken with the VNA. Note that the radio and VNA results are within roughly 0.5 dB of each other. The noise floor of the radio, as shown by the lower plot measured with terminated Tx and Rx ports, is near -100 dB. The radio is capable of 70 frequency sweeps per second for 100 points between 17.5 GHz and 26.5 GHz (~10 times the speed of the VNA).


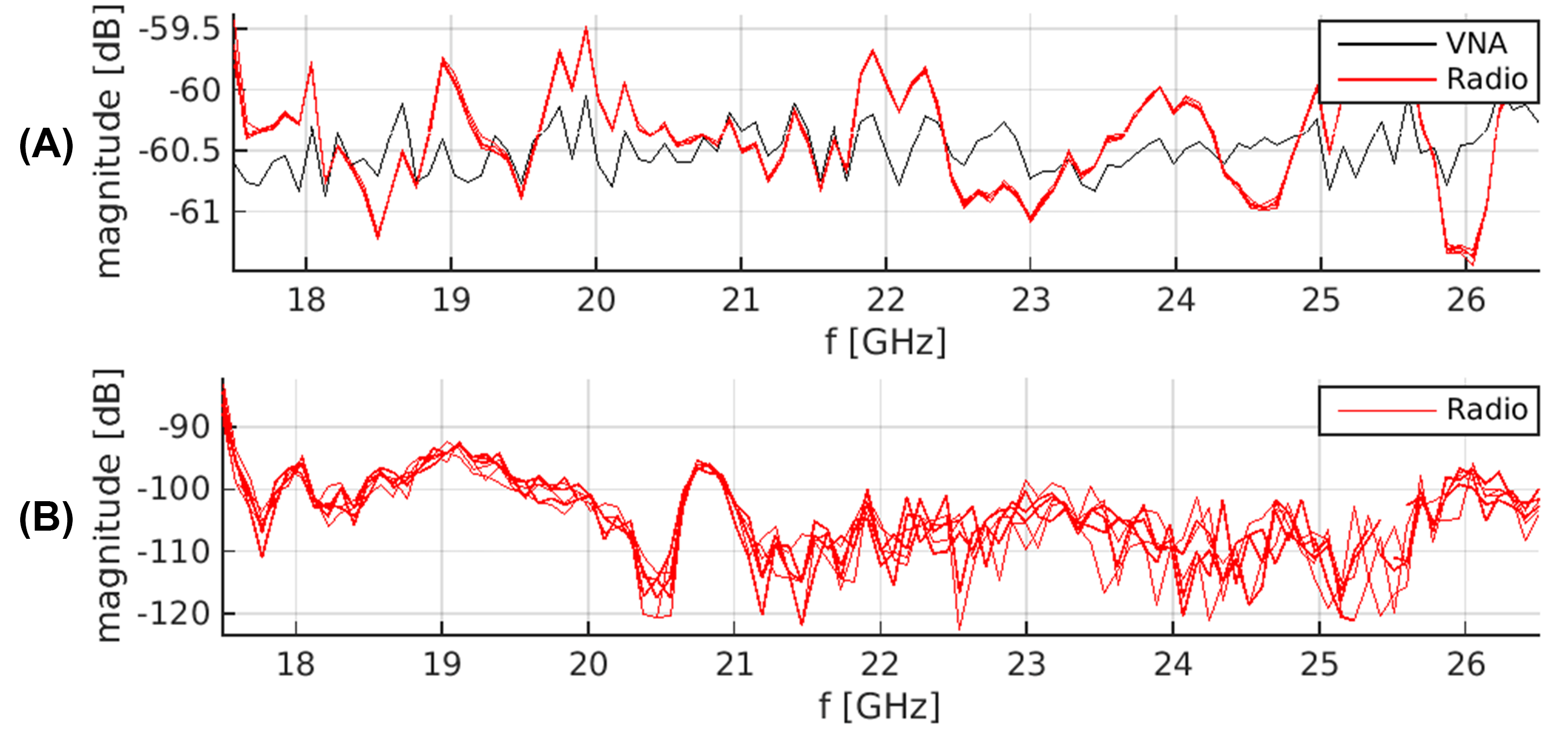


***Fig. S9.*** *(A) Comparison of through measurement (60 dB ATT.) results between radio and an Agilent N5227A network analyzer and (B) noise floor of radio with terminate Tx and Rx ports attached to a matched load (50 Ω).*

**Calibration**

This section describes the method utilized for calibrating the feeding network.


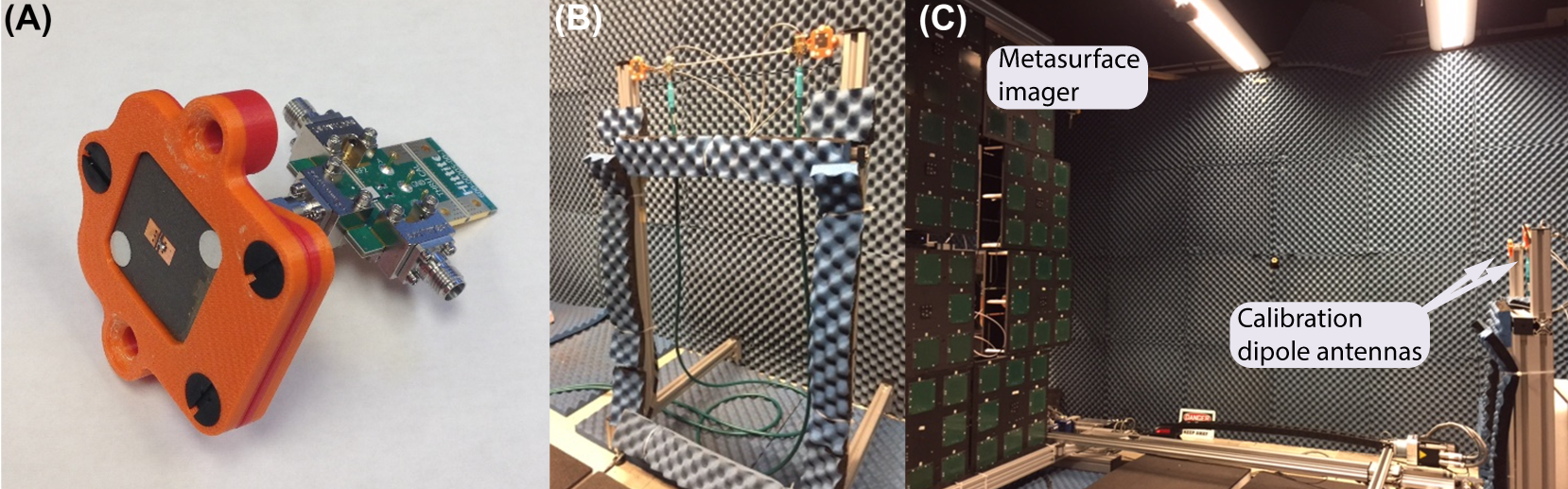


***Fig. S10.*** *(A) The dipole antennas used in the calibration. (B) The in-scene calibration antennas and mount. (C) View of imaging system and calibration antennas from behind the imaging scene.*

The phase advance through the feeding network must be removed from the measurements to accurately correlate their phase and construct the measurement matrix, H. Because the feeding network contains switches and semi-rigid components, it is not possible to directly measure the response without perturbing the measurements. Furthermore, the number of paths to be calibrated is on the order of 1728, making direct measurement prohibitively time consuming. Instead, two printed dipole antennas, with a wideband response across the K-band, are placed at the back of the imaging scene, as shown in Fig. S10. One is connected to the Tx distribution chain by an extended low-loss cable and one is connected to the Rx distribution chain, also via a long low-loss cable. This allows direct measurements between the Tx and Rx dipole antennas to the metasurface panels. Further, a direct path through the connecting cables can be measured, via a set of electronic bypass switches. This allows any drift in the measurements, due to the long feeding cables, to be calibrated out. The remaining small phase advance due to the antennas and radiation pattern of the dipole is stable and has been characterized using a NFS. To perform a calibration, measurements of the free space and feeding network path are obtained. The exact positions of the antennas and panels are determined using stereophotogrammetry equipment (CREAform, MaxShot). The free space phase advance from all the Tx panels to the Rx dipole antennas, and all the Rx panels to the Tx dipole antenna can then be obtained by modeling the position of the panels and dipole antennas (and their NFS properties). These signals can then be cross-correlated to determine the phase advance in the feeding network for each Tx/Rx path, and hence, the necessary correction terms. This hardware setup provides excellent SNR which is important given the large dynamic range of the radiation measurements for a frequency-diverse metasurface panel. Further this calibration can be performed periodically to counter any drift in the system (assuming the system is mechanical stable). We note that while the calibration dipoles are not used (during imaging process), the bypass switch networks connect the dipole antennas to a termination, rendering them non-scattering and hence they will not perturb the imaging process. As a result, the calibration stage can remain in the scene continuously. Finally, we note that without calibration no image formation is possible as shown in Fig. S11; inside the region selected by the structured light sensor (as discussed below) we only see reconstructed noise.


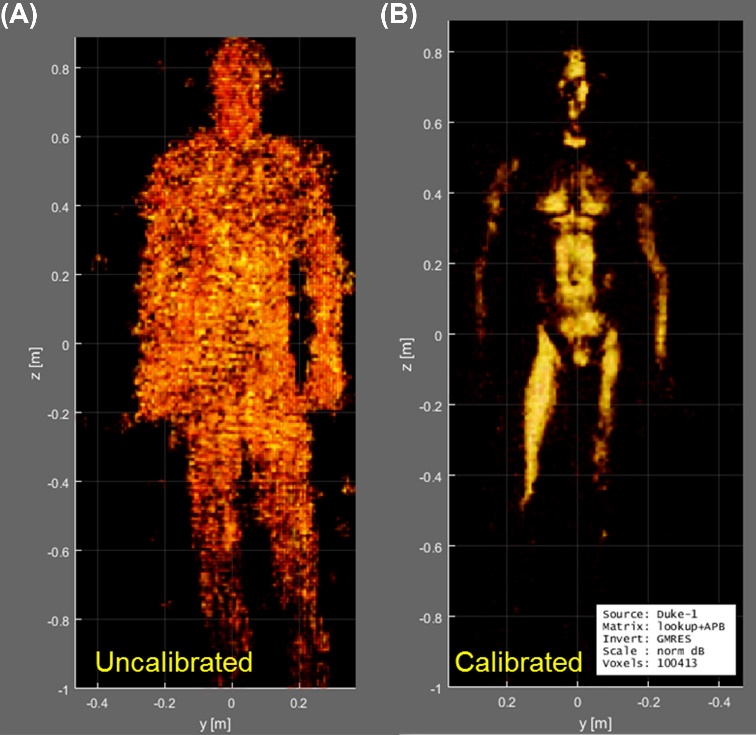


***Fig. S11.*** *Reconstruction without (A) calibration implemented and (B) with calibration.*

**Image Reconstruction**

This section discusses the reconstruction process.

**
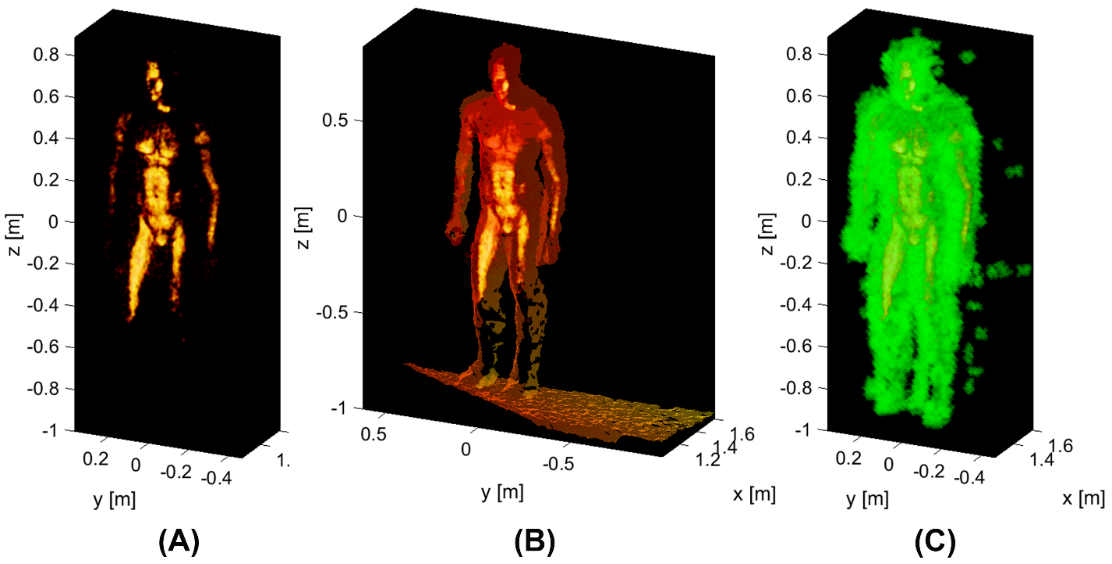
**

***Fig. S12.*** *(A) RF reconstruction of a mannequin target. (B) Kinect optical surface detection (and RF reconstruction). (C) RF Region of Interest (ROI) selected for reconstruction utilizing the Kinect surface as a reference (and RF reconstruction).*

Two structured light sensor (Microsoft Kinect) are used to approximately determine the optical surface of the target in the scene. This surface, in turn, is used to define a larger envelope, or Region of Interest (ROI), containing the optically observable target to be reconstructed in the RF regime (this includes a buffer thickness of 8 cm, +/-4 cm from the optical surface, as shown in Fig. S12). The Kinects are placed on opposite sides of the aperture and their measurements are combined to ensure that a wide viewing angle and ROI volume containing the target is captured. Jitter motors attached to each Kinect prevent direct interference between the two sensors by blurring out the projected IR pattern of the non-relevant sensor. The Kinects are connected to the workstation and are controlled via Matlab using the OpenKinect open source libraries. The Kinect provide an inexpensive means of selecting the pertinent ROI for reconstruction while reducing computation load, hence generally improving imaging rates.

We note that even when extracting an ROI for a mannequin target utilizing the IR sensor, the size of $\mathbf{f}$ will typically correspond to between 100,000-200,000 voxels, at Nyquist sampling (cross-range: 0.7 cm; range: 1.6 cm). The measurement matrix for the full maximum unambiguous range, **H**, will have over 34 million elements and require ~130 GBs of storage in single precision floating point format. While it is conceivable that this much data could be stored in random access memory on a top of the line workstation, it will be taxing to manage and access quickly. Instead, we calculate **H** “on the fly” on a GPU (NVIDIA, K5000) running compiled CUDA code. As discussed in the main text, the imaging equation cannot be directly inverted, since **H** does not possess an inverse---as the number of measurements generally exceeds the number of voxels being sampled. The adjoint operation provides a basic estimation of the scene, with **f**=**H*****g** (where **H*** is the conjugate transpose of **H**) and requires only a single matrix multiplication. This constitutes the fastest reconstruction, as shown in Fig. S13(A-C), but not necessarily the most accurate. Alternatively, a Least square (LS) such as GMRES can be used. It is an iterative approach that seeks to minimize the objective function$\text{arg min}_{\text{f}}\text{||}\mathbf{g}-\mathbf{Hf}||_{2}^{2}+\lambda R(\mathbf{f})$, for $\mathbf{f}$. The $\lambda R(\mathbf{f})$ term is an appropriate regularizer that speeds/ensures convergence. Fig. S13(D-F) shows the least square reconstruction. The LS algorithm extracts additional information from the non-orthogonal measurements and improves image resolution.

**
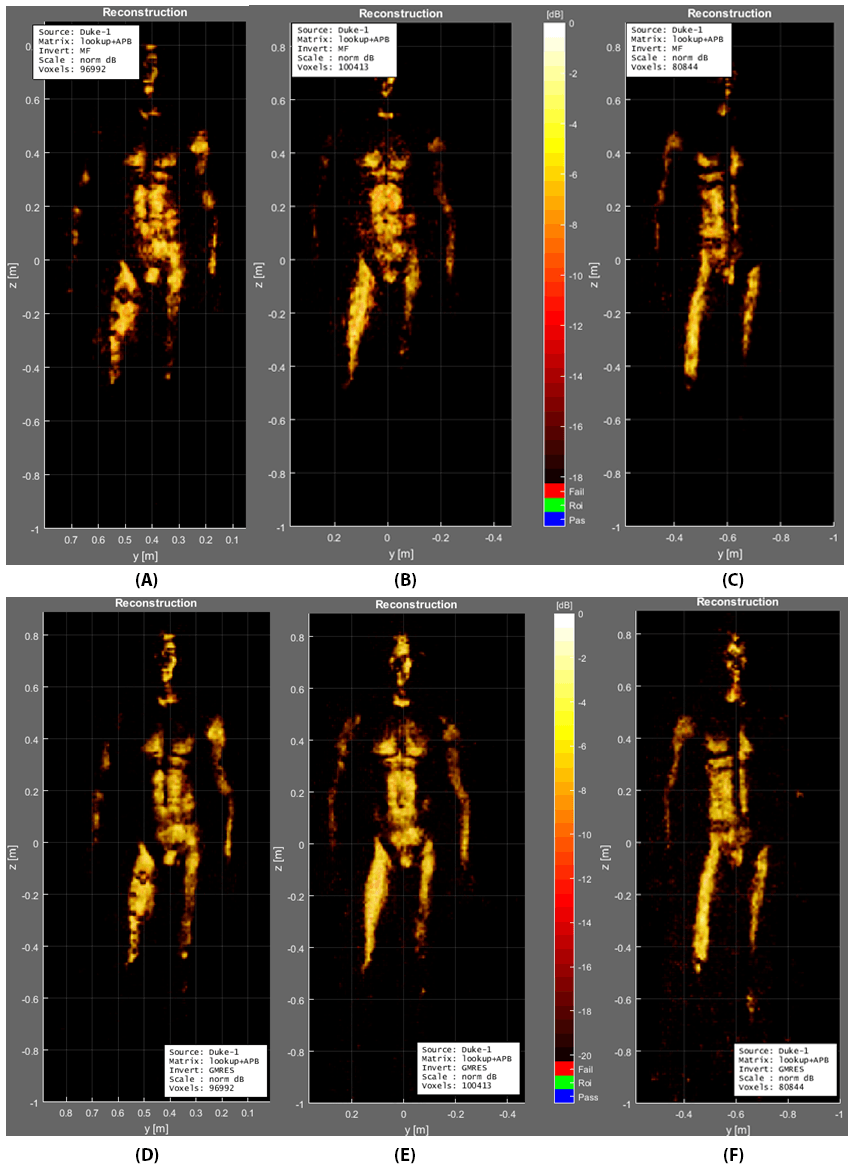
**

***Fig. S13.*** *(A-C) A Matched filter reconstruction for a Mannequin in various cross-range positions in the scene (as shown in the paper). (D-F) A Least Squares reconstruction using the same measurement data as (A-C).*

**Image Stitching**

This section discusses the composite image reconstructed from multiple specular images.

The mirror-like specular reflection of microwave radiation from metals and materials with high water content limits the total amount of coverage any single image of a target in the scene can recover. However, multiple images of a target, from multiple locations and orientations, can be combined, or stitched, to produce a composite image with better coverage. To obtain the stitched images shown in Movie S14, a mannequin was placed on a rotation stage and imaged at regular 5 degree angular intervals. Photogrammetry was used to optically measure the position of the rotation stage, and an orthogonal Procrustes method was applied to deduce the location and orientation of the stage’s rotational axis.  The known angle revolved for each image was then employed to construct an affine transformation that inverts the rotation introduced by the stage, effectively aligning the images on top of each other.  The separate images were resampled on a common grid and incoherently added together to form a final stitched image. The included movie shows the stitched 3D image. Body pattern recognition (increasingly available in low cost optical sensors, such as the Microsoft Kinect One) would be a natural evolutionary approach to practically deploy these stitching techniques.

**Movie S14.** Multiple coherently reconstructed images of a mannequin, rotated through 5 degree increments, are combined to produce a composite image that captures the detailed structure of the target.
